# Case Report: Concurrent ventricular septal defect, pulmonic stenosis, and intrapulmonary arteriovenous anastomoses in a Korean raccoon dog (*Nyctereutes procyonoides koreensis*)

**DOI:** 10.3389/fvets.2026.1969910

**Published:** 2026-10-07

**Authors:** Seoyeon Kim, Myeongsu Kim, Jae-Ik Han, Kichang Lee, Hakyoung Yoon

**Affiliations:** 1 Veterinary Medical Imaging, College of Veterinary Medicine, Jeonbuk National University, Iksan, Republic of Korea; 2 Laboratory of Exotic and Wild Animal Medicine, College of Veterinary Medicine, Jeonbuk National University, Iksan, Republic of Korea; 3 Jeonbuk Wildlife Center, Jeonbuk National University, Iksan, Republic of Korea

**Keywords:** bidirectional shunt, intrapulmonary arteriovenous anastomoses, *Nyctereutes procyonoides*, pulmonic stenosis, ventricular septal defect

## Abstract

A 3-month-old intact male Korean raccoon dog (*Nyctereutes procyonoides koreensis*) was rescued and admitted to the Jeonbuk Wildlife Rescue Center with lethargy, dehydration, and dermatologic lesions consistent with *Sarcoptes scabiei* infestation. Physical examination detected a grade IV/VI systolic murmur, and thoracic radiography revealed generalized cardiomegaly. Transthoracic echocardiography identified a perimembranous ventricular septal defect (VSD) with left-to-right shunting. Although no distinct structural abnormalities of the pulmonic valve were observed, increased pulmonic flow velocity (3.87 m/s), mild pulmonic regurgitation (1.68 m/s), and post-stenotic dilation of the main pulmonary artery supported a diagnosis of pulmonic stenosis (PS). Agitated saline contrast echocardiography demonstrated intrapulmonary arteriovenous anastomoses (IPAVAs), with microbubbles appearing in the left heart after the fourth cardiac cycle; this shunting decreased following oxygen supplementation. Five weeks later, thoracic radiography demonstrated a reduction in cardiac silhouette size, although cardiomegaly persisted. Echocardiography demonstrated progression of the VSD to bidirectional shunting. Systolic doming of the pulmonic valve, together with increased pulmonic flow velocity (4.39 m/s) and pulmonic regurgitation velocity (2.07 m/s), indicated worsening PS. Repeat agitated saline contrast echocardiography revealed microbubbles in the left heart within four cardiac cycles and in the abdominal aorta, indicating a right-to-left shunt component consistent with bidirectional shunting. These findings were attenuated after oxygen supplementation. The Korean raccoon dog subsequently died during anesthetic induction for further diagnostic imaging. Postmortem examination confirmed a perimembranous VSD and type A valvular PS characterized by fused commissures, with concurrent right ventricular hypertrophy, dilation, and interventricular septal thickening. To our knowledge, this case report is the first to describe the concurrence of VSD, PS, and IPAVAs in a Korean raccoon dog. It suggests that progressive PS may contribute to reversal of shunting across a VSD.

## 1 Introduction

The Korean raccoon dog (*Nyctereutes procyonoides koreensis*) is a wild canid commonly observed in Korea and a member of the family *Canidae* (1). In domestic dogs, ventricular septal defect (VSD) and pulmonic stenosis (PS) are relatively common congenital heart defects, although their concurrent occurrence is rare (2). To our knowledge, atrial septal defect (ASD) is the only congenital cardiac defect previously reported in Korean raccoon dogs, and neither VSD nor PS has been described in this subspecies (3).

VSD is a congenital defect of the interventricular septum resulting from abnormal septal development during embryogenesis. Perimembranous VSD is the most common subtype reported in dogs (4, 5). In general, the higher pressure in the left ventricle produces a left-to-right shunt across the defect (4, 6). However, bidirectional or reversed (right-to-left) shunting may occur when the defect is large or when right ventricular pressure is elevated, such as in the presence of PS or pulmonary hypertension (4, 6–8).

PS is a congenital cardiac defect caused by dysplasia of the semilunar valve between the right ventricle (RV) and the pulmonary artery, with valvular form representing the most common subtype in dogs (9–11). The resulting obstruction to right ventricular outflow increases right ventricular systolic pressure and imposes pressure overload on the RV (4).

When PS and VSD coexist, the increase in right ventricular pressure caused by PS may reverse the interventricular pressure gradient (PG), resulting in a right-to-left shunt even in the absence of pulmonary vascular disease (4, 12). This mechanism differs from Eisenmenger syndrome, in which shunt reversal results from progressive pulmonary hypertension (4, 8).

Intrapulmonary arteriovenous anastomoses (IPAVAs) are vascular pathways that connect the pulmonary arterial and venous circulations, bypassing the pulmonary capillary network. Although their physiological role remains incompletely understood, IPAVAs are considered hypoxia-responsive pathways that recruit under hypoxic conditions and close following oxygen supplementation (13, 14). They can be detected using agitated saline contrast echocardiography (bubble study) (13, 14). During a bubble study, the appearance of microbubbles in the left heart within four cardiac cycles indicates an intracardiac right-to-left shunt, whereas their appearance after the fourth cardiac cycle suggests an extracardiac shunt, such as IPAVA (13, 15). A previous study reported a 75% prevalence of IPAVAs at rest in healthy Korean raccoon dogs (13).

This case report describes the concurrent occurrence of perimembranous VSD, valvular PS, and IPAVAs in a Korean raccoon dog, diagnosed using echocardiography, agitated saline contrast echocardiography, and postmortem examination.

## 2 Case description

An intact male Korean raccoon dog (*Nyctereutes procyonoides koreensis*), estimated to be 3 months old and weighing 1.2 kg, was rescued and admitted to the Jeonbuk Wildlife Rescue Center. On presentation, the animal exhibited lethargy, dehydration, generalized alopecia, and hyperkeratosis consistent with severe *S. scabiei* infestation. Physical examination revealed a grade IV/VI systolic murmur, and the systolic blood pressure was 120 mmHg. Complete blood count (CBC) demonstrated anemia (hematocrit, 25.3%; reference range, 33–47%) and leukocytosis (white blood cell count, 23.6 × 10^9^/L; reference range, 6.15–13.65 × 10^9^/L), findings considered secondary to the concurrent *S. scabiei* infestation (16).

Thoracic radiography (HF-525 PLUS, Ecoray, Seoul, Korea) revealed generalized cardiomegaly, with a vertebral heart score (VHS) of 12.2, a vertebral left atrial score (VLAS) of 2.8 (Figure 1A), and a cardiothoracic ratio (CTR) of 82% (Figure 1B). All measurements exceeded the previously reported reference values for healthy Korean raccoon dogs (VHS, 9.12 ± 0.74; VLAS, 1.50 ± 0.31; CTR, 0.69 ± 0.07) (17).

**Figure 1:**
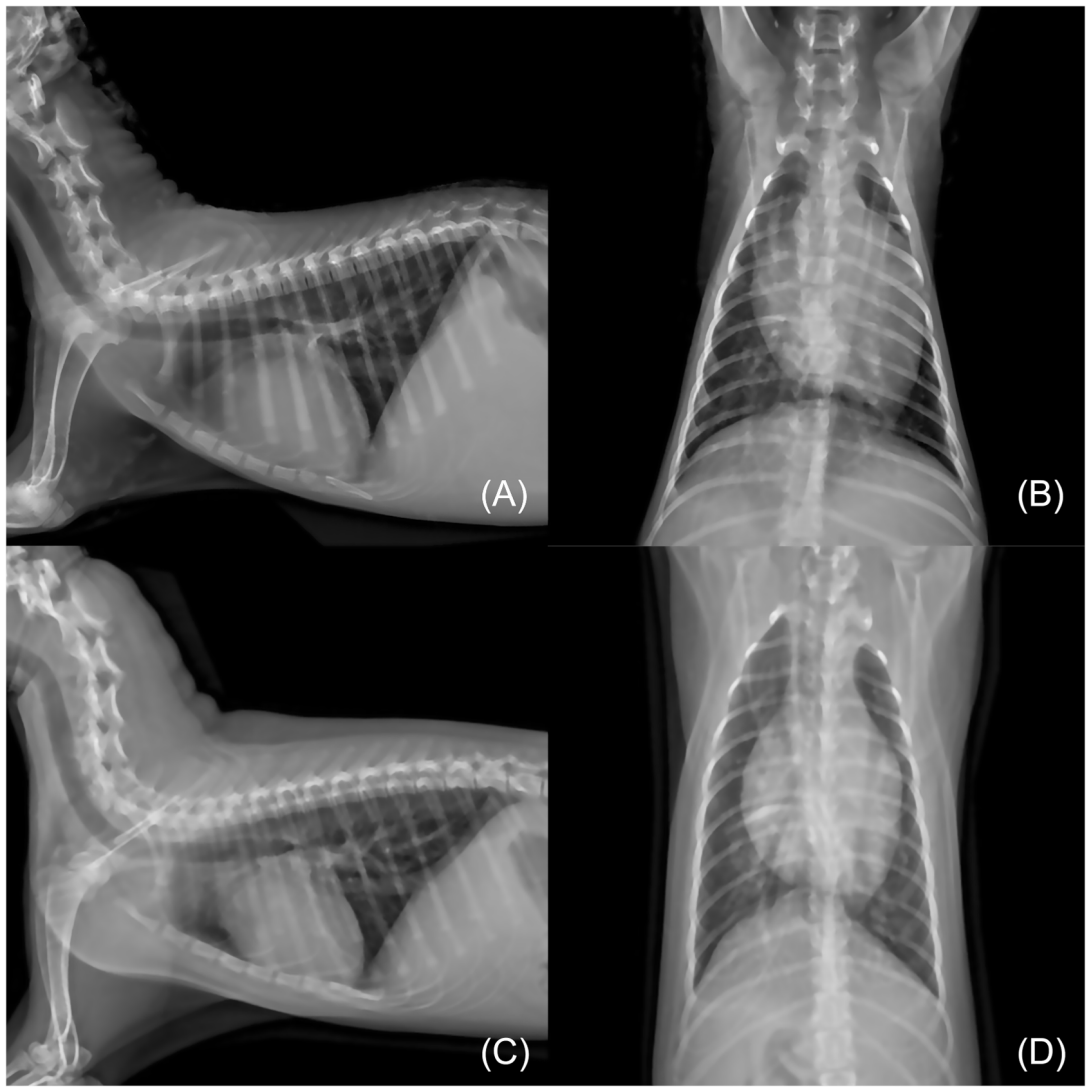
Thoracic radiographs obtained at the initial examination demonstrated increased VHS (12.2) and VLAS (2.8) on the right lateral view **(A)**, and an increased CTR (82%) on the ventrodorsal view **(B)**. At 5-week follow-up, these indices had decreased; however, the VHS (9.86) remained at the upper limit of normal, the VLAS (2.1) remained increased on the right lateral view **(C)**, and the CTR (78%) remained mildly increased on the ventrodorsal view **(D)**. Generalized cardiomegaly was evident at both examinations.

Transthoracic echocardiography was performed without sedation or anesthesia using a 5-MHz transducer (Aplio i800, Canon Medical Systems, Tokyo, Japan). The heart rate was mildly elevated at 161 beats/min compared with the reported reference range for raccoons (94–134 beats/min) (18). B-mode imaging from the right parasternal five-chamber long-axis view identified a 3.24-mm perimembranous VSD located between the subaortic region and the RV (Figure 2A). Color Doppler and continuous-wave Doppler imaging demonstrated a left-to-right shunt from the left ventricular outflow tract (LVOT) to the RV during early systole, with a peak velocity of 2.32 m/s (PG: 21.5 mmHg) (Figure 2C).

**Figure 2:**
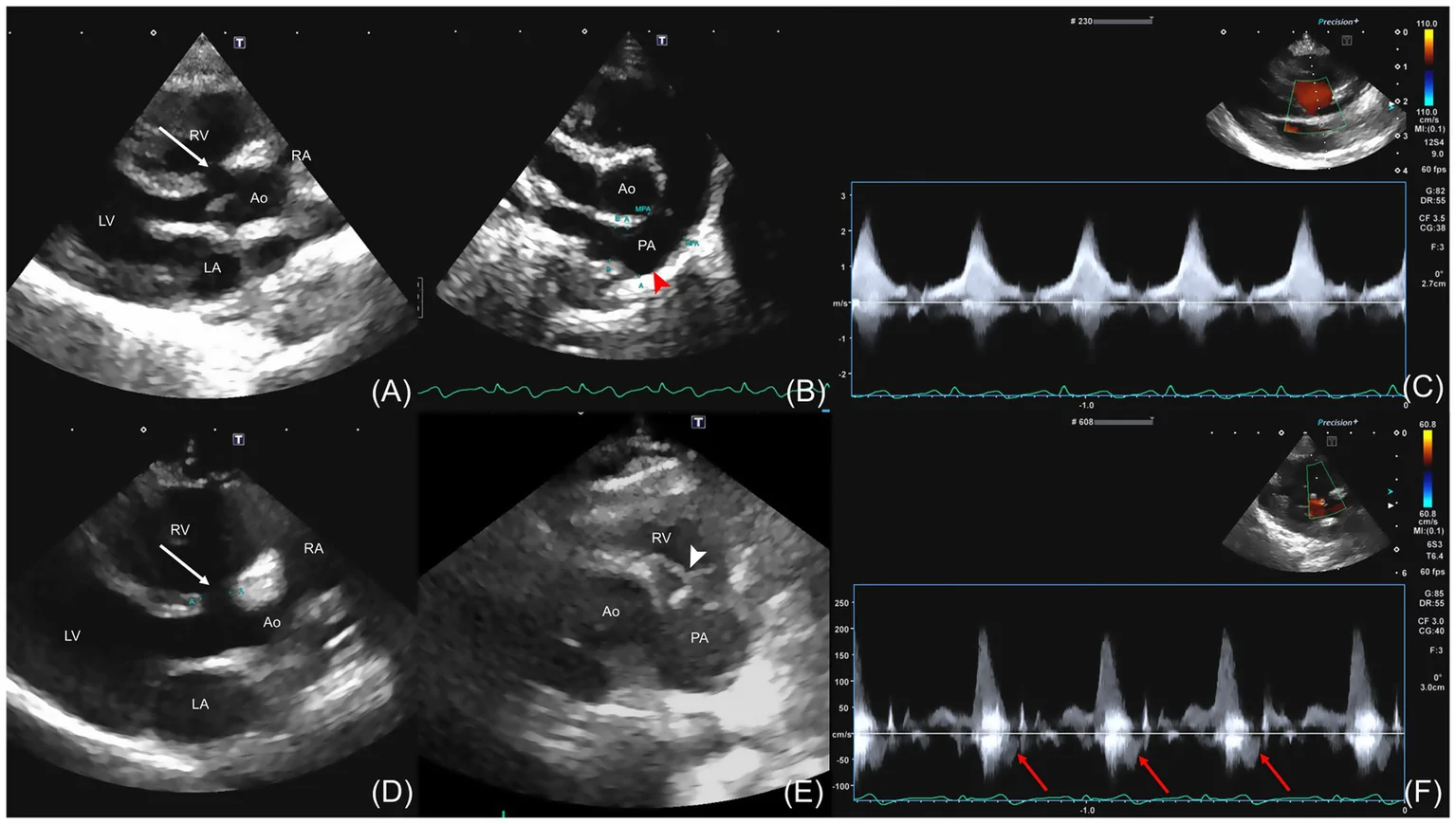
Echocardiographic findings at the initial examination **(A–C)** and the 5-week follow-up **(D–F)**. In the right parasternal five-chamber long-axis view, a perimembranous ventricular septal defect measuring 3.24 mm at the initial examination [**(A)**, white arrow] and 4.2 mm at the 5-week follow-up [**(D)**, white arrow] was identified. In the right parasternal short-axis view at the heart base level, poststenotic dilation of the pulmonary artery was observed at the initial examination [**(B)**, red arrowhead], although the pulmonic valve was not clearly visualized. In contrast, at the 5-week follow-up, a dome-shaped pulmonic valve was observed during systole [**(E)**, white arrowhead]. Color Doppler and continuous-wave Doppler imaging demonstrated a left-to-right shunt across the ventricular septal defect at the initial examination **(C)**, whereas bidirectional shunting, including an early-diastolic right-to-left shunt [**(F)**, red arrow], was observed at the 5-week follow-up. LA, left atrium; LV, left ventricle; Ao, aorta; RA, right atrium; RV, right ventricle; PA, pulmonary artery.

In the right parasternal short-axis view at the heart base level, no discrete site of stenosis was identified; however, marked dilation of the pulmonary artery distal to the annulus was observed, consistent with post-stenotic dilation (Figure 2B). Continuous-wave Doppler revealed an increased pulmonic valve velocity of 3.87 m/s (PG: 59.9 mmHg) during late systole. Mild pulmonary regurgitation was also identified at 1.68 m/s (PG: 11.2 mmHg). Collectively, these findings were consistent with PS. The aortic-to-pulmonary annulus ratio was 1.06 (aortic annulus: 7.0 mm; pulmonary annulus: 6.6 mm), excluding pulmonic valve annular hypoplasia (>1.2) (10).

The left atrial-to-aortic root ratio (LA/Ao) was 1.55, indicating mild left atrial enlargement relative to the reported reference value (1.22 ± 0.14) (17). Tissue Doppler imaging demonstrated an increased E/E′ ratio of 25.72, suggestive of elevated left ventricular filling pressure. The tricuspid annular plane systolic excursion (TAPSE) was 0.64 cm, indicating reduced RV systolic function (17). Echocardiography also revealed right ventricular free wall hypertrophy, right ventricular dilation, and interventricular septal flattening. Tricuspid regurgitation was not detected. The right pulmonary artery distensibility (RPAD) index was 53%, which was within the reported reference range (17). The pulmonary-to-systemic blood flow ratio (Qp/Qs) was not calculated as concurrent PS precludes reliable estimation (4).

A bubble study was performed from the left parasternal apical four-chamber view to evaluate shunt direction and detect IPAVAs. Agitated saline (2 mL) was rapidly injected through the cephalic vein, and bubble scores were assigned according to a previously described 0–5 grading system based on the maximum number and distribution of microbubbles observed in the left ventricle in a single frame. The scoring criteria were as follows: bubble score 0, no microbubbles; bubble score 1, 1–3 microbubbles; bubble score 2, 4–12 microbubbles; bubble score 3, >12 microbubbles appearing as a bolus; bubble score 4, >12 microbubbles heterogeneously distributed throughout the left ventricle; and bubble score 5, >12 microbubbles homogeneously distributed throughout the left ventricle (13, 14). No microbubbles were detected in the left heart during the first four cardiac cycles (bubble score 0, Figure 3A). After the fourth cardiac cycle, numerous heterogeneously distributed microbubbles appeared throughout the left ventricle (bubble score 4, Figure 3B), consistent with the presence of IPAVAs. Subsequently, 100% oxygen was administered via a mask at a flow rate of 5 L/min for 5 min, after which a repeat bubble study was performed (13). Following oxygen supplementation, the number of microbubbles observed after the fourth cardiac cycle decreased to bubble score 2 (Figure 3C), indicating reduced shunt flow through the IPAVAs. No microbubbles were detected in the abdominal aorta before or after oxygen supplementation.

**Figure 3:**
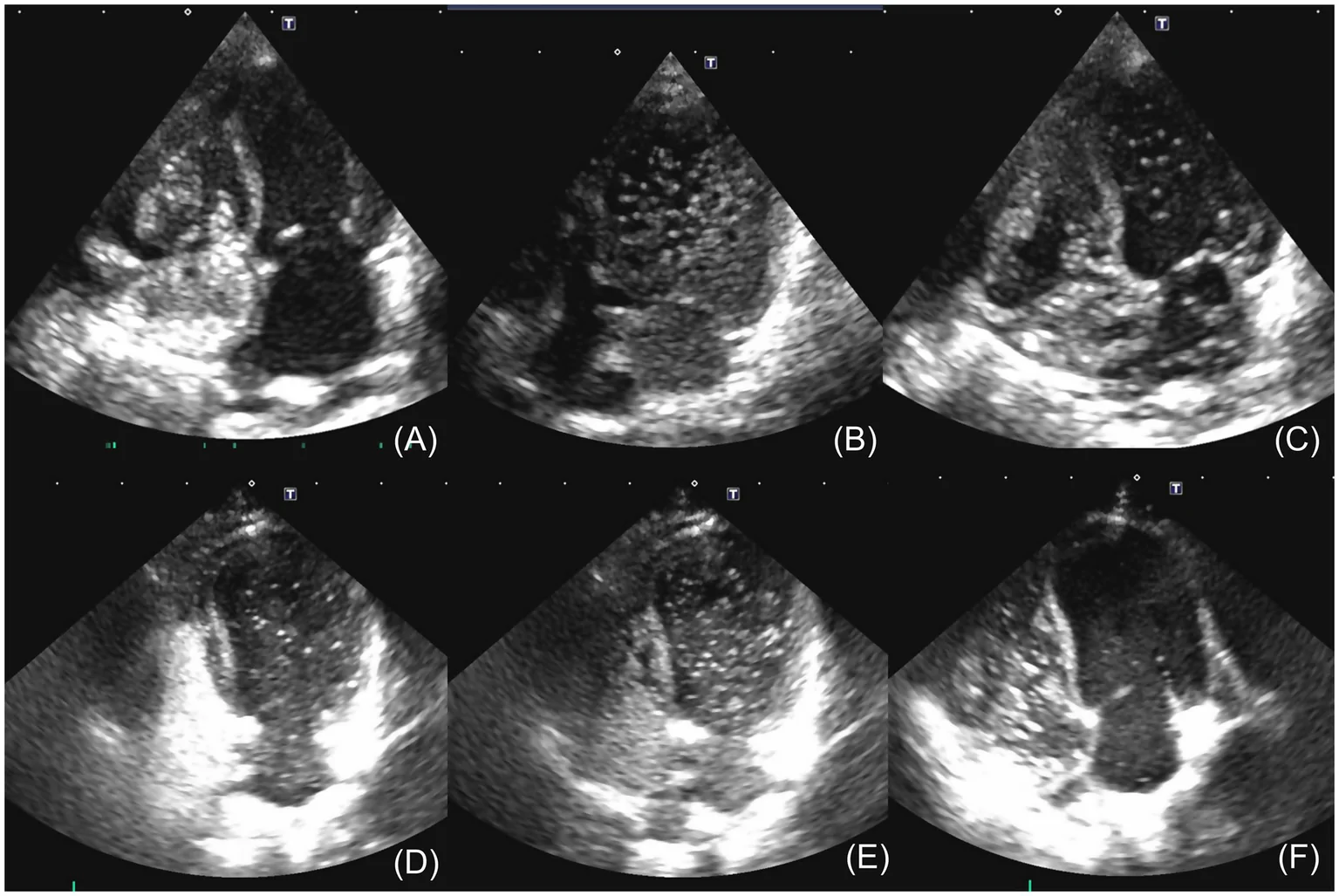
Agitated saline contrast echocardiography (bubble study) performed at the initial examination **(A–C)** and the 5-week follow-up **(D–F)**. Before oxygen supplementation, no microbubbles were detected in the left heart within the first four cardiac cycles at the initial examination (bubble score 0) **(A)**, whereas 4–12 microbubbles were observed within the first four cardiac cycles at the 5-week follow-up (bubble score 2) **(D)**. This finding suggests the presence of an intracardiac right-to-left shunt. At the initial diagnosis, after the fourth cardiac cycle and pre-oxygen supplementation, more than 12 microbubbles were heterogeneously distributed throughout the left ventricle (bubble score 4) **(B)**, and this number decreased to 4–12 microbubbles (bubble score 2) after oxygen supplementation **(C)**. At the 5-week follow-up, more than 12 heterogeneously distributed microbubbles remained visible after the fourth cardiac cycle before oxygen supplementation (bubble score 4), although fewer were observed than at the initial examination **(E)**. Following oxygen supplementation, the number further decreased to 1–3 microbubbles (bubble score 1) **(F)**.

Because standardized guidelines for the medical management of cardiac disease in Korean raccoon dogs are unavailable and the animal showed no overt cardiovascular clinical signs, cardiac pharmacotherapy was not initiated. Oral ferrous sulfate was administered to treat anemia. Ivermectin, meloxicam, metronidazole, and doxycycline were administered to treat the *S. scabiei* infestation and secondary bacterial infections, and fluid therapy was provided to correct dehydration.

At the 5-week re-examination, dermatologic lesions associated with the *S. scabiei* infestation, along with systemic signs including lethargy and dehydration, had markedly improved. CBC demonstrated improvement in anemia and leukocytosis. On physical examination, a grade IV/VI systolic murmur persisted, and the heart rate had decreased to 140 beats/min. Thoracic radiography revealed a VHS of 9.86, a VLAS of 2.1, and a CTR of 78% (Figures 1C,D). Although these indices had decreased compared with the initial examination, VHS remained at the upper limits of normal, whereas VLAS and CTR remained mildly increased (17). Transthoracic echocardiography from the right parasternal five-chamber long-axis view identified a perimembranous VSD measuring 4.2 mm, representing an increase from the initial measurement of 3.24 mm (Figure 2D). Color Doppler and continuous-wave Doppler demonstrated a left-to-right shunt during systole; however, an additional right-to-left shunt from the RV to the LVOT was observed during early diastole, resulting in a bidirectional shunt pattern. The peak velocity of the systolic left-to-right shunt was 1.81 m/s (PG: 13.1 mmHg), whereas the early-diastolic right-to-left shunt had a peak velocity of 0.67 m/s (PG: 1.79 mmHg) (Figure 2F).

In the right parasternal short-axis view at the heart base level, systolic doming of the pulmonic valve with minimal leaflet thickening was newly identified (Figure 2E). Continuous-wave Doppler demonstrated an increased pulmonic valve peak velocity of 4.39 m/s during late systole (PG: 77.0 mmHg). The peak velocity of pulmonic regurgitation also increased to 2.07 m/s (PG: 17.1 mmHg), compared with the initial examination. The aortic-to-pulmonary annulus ratio was 0.96 (aortic annulus: 8.3 mm; pulmonary annulus: 8.6 mm), similar to the initial measurement. Based on the echocardiographic findings, the PS was considered consistent with type A valvular PS.

The LA/Ao ratio decreased to 0.94, indicating resolution of left atrial enlargement. Tissue Doppler imaging demonstrated a reduced E/E′ ratio of 16.08, suggesting a decrease in left ventricular filling pressure. The TAPSE decreased to 0.48 cm, suggesting a further reduction in RV systolic function (17). Mild progression of right ventricular free wall hypertrophy, right ventricular dilation, and interventricular septal flattening was observed compared with the previous examination. Tricuspid regurgitation was not detected. The RPAD index was 43%, remaining within the reported reference range (17). Collectively, these echocardiographic findings supported the diagnosis of a complex congenital cardiac anomaly comprising VSD and PS.

Before oxygen supplementation, 4–12 microbubbles were observed in the left ventricle within the first four cardiac cycles (bubble score 2, Figure 3D), indicating an intracardiac right-to-left shunt. After the fourth cardiac cycle, microbubbles remained heterogeneously distributed throughout the left ventricle despite being slightly reduced in number compared with the initial examination (bubble score 4, Figure 3E), consistent with persistent IPAVAs. Subsequently, the same oxygen supplementation protocol used during the initial examination was applied. Following oxygen supplementation, no microbubbles were detected within the first four cardiac cycles (bubble score 0), and only a few bubbles were observed after the fourth cardiac cycle (bubble score 1, Figure 3F). Microbubbles were present in the abdominal aorta before oxygen supplementation but were absent thereafter.

Computed tomography (CT) was planned to further characterize the cardiac anatomy, including the VSD and PS. Medetomidine (0.05 mg/kg, IV) and alfaxalone (2.2 mg/kg, IV) were administered as premedication and induction agents, respectively. During induction, however, the Korean raccoon dog developed severe arrhythmia and died. Postmortem examination confirmed a 4-mm perimembranous VSD located between the LVOT and RV (Figure 4A). Type A valvular PS was also identified, with mild leaflet thickening and commissural fusion of the intermediate, right, and left cusps. In addition, right ventricular free wall hypertrophy, interventricular septal hypertrophy (Figure 4B), and right ventricular dilation were observed. On histopathology, hematoxylin and eosin (H&E) and Masson’s trichrome staining of the pulmonary valve demonstrated leaflet hypertrophy, fibrosis, and degeneration.

**Figure 4:**
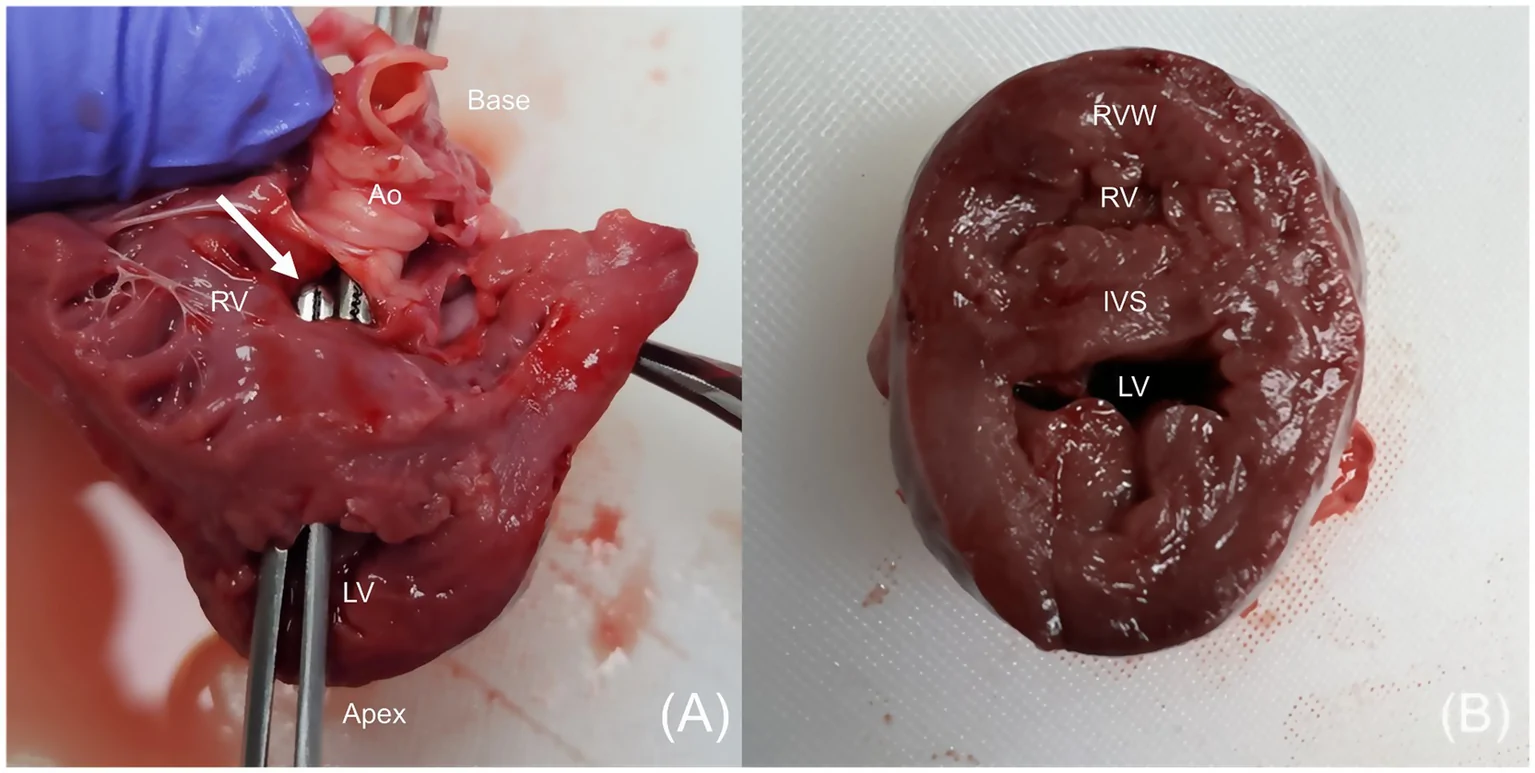
Gross pathological findings at necropsy. **(A)** Longitudinal section of the heart demonstrating a perimembranous ventricular septal defect (white arrow). **(B)** Transverse section of the heart demonstrating right ventricular free wall hypertrophy and interventricular septal hypertrophy. Ao, aorta; LV, left ventricle; RV, right ventricle; RVW, right ventricular wall; IVS, interventricular septum.

## 3 Discussion

The clinical significance of VSD is primarily determined by defect size and the magnitude of shunt flow (19). Small restrictive VSDs are typically hemodynamically insignificant and do not cause substantial volume overload of the heart or pulmonary circulation. By contrast, large nonrestrictive VSDs increase pulmonary blood flow, which may result in pulmonary hypertension secondary to chronic left-to-right shunting (7, 19). As pulmonary arterial pressure approaches or exceeds systemic arterial pressure, the shunt may become bidirectional or reverse to right-to-left, representing progression to Eisenmenger syndrome (4, 7).

At the 5-week follow-up, the appearance of bidirectional shunting through the VSD raised the possibility of pulmonary hypertension as a differential diagnosis (7). However, RPAD index remained within the reported reference range. Although post-stenotic dilation of the pulmonary artery associated with PS was present, generalized pulmonary arterial enlargement suggestive of pulmonary hypertension was not observed. Pulmonic regurgitation also remained mild. These findings did not provide sufficient evidence of pulmonary hypertension (4, 7, 19, 20). Therefore, the bidirectional shunt in this case was considered more likely to be associated with increased right ventricular pressure secondary to pulmonic stenosis rather than Eisenmenger physiology caused by progressive pulmonary vascular disease.

Tetralogy of Fallot was also considered as a differential diagnosis, as the coexistence of VSD, right ventricular outflow tract obstruction, and right ventricular hypertrophy overlapped with its characteristic features. However, an overriding aorta was not identified on either echocardiographic or postmortem examination, thereby excluding tetralogy of Fallot (21, 22).

At the 5-week follow-up, systolic doming of the pulmonic valve with minimal leaflet thickening was newly identified, accompanied by increases in pulmonic valve velocity and PG, as well as an increase in pulmonic regurgitation. Although PS is generally considered a congenital and fixed valvular stenosis in dogs, progression during growth has been reported in individual puppies with mild PS (23). The changes observed in this case may therefore represent progression of valvular PS, although the underlying mechanism remains unclear. Progressive right ventricular outflow tract obstruction may have increased right ventricular pressure, thereby reducing the interventricular PG across the VSD and contributing to the development of bidirectional shunting (24). The resulting reduction in left-to-right shunt flow may have decreased pulmonary blood flow and partially alleviated left-sided volume overload. This may also explain the decreases in VHS, VLAS, and LA/Ao observed at follow-up (12, 24). The hemodynamic interaction between concurrent PS and VSD may be comparable to that achieved by pulmonary artery banding, which limits excessive left-to-right shunting through a VSD (12). By increasing right ventricular pressure, both conditions reduce left-to-right shunt flow and pulmonary blood flow, thereby decreasing volume overload of the pulmonary circulation and left heart. The absence of clinical signs of left-sided heart failure throughout the approximately 5-week follow-up period suggests that a relatively balanced hemodynamic state was maintained (4, 12, 24).

During the initial agitated saline contrast study, microbubbles appeared in the left heart only after the fourth cardiac cycle, consistent with the presence of IPAVAs. Following oxygen supplementation, the number of microbubbles appearing after the fourth cardiac cycle decreased. This finding is consistent with previous studies demonstrating that IPAVAs dynamically open under hypoxic conditions and close in response to increased oxygen availability, resulting in reduced IPAVA shunt flow (13, 14). At the 5-week follow-up, fewer microbubbles were observed after the fourth cardiac cycle than at the initial examination, suggesting reduced IPAVA patency during postnatal growth. This observation is consistent with studies reporting postnatal regression of IPAVAs in humans and lambs (25, 26). Although pulmonary arteriovenous malformation (PAVM) could not be definitively excluded as CT angiography was not performed, the oxygen-dependent reduction in microbubble passage and the apparent postnatal regression of shunt flow were more consistent with functional IPAVAs than with a fixed structural PAVM (14).

Unlike the initial examination, the follow-up agitated saline study demonstrated microbubbles in the left heart within the first four cardiac cycles and in the abdominal aorta. These findings indicate right-to-left shunting through the VSD in addition to the existing left-to-right shunt, consistent with bidirectional shunting (13). Following oxygen supplementation, microbubbles were no longer detected within the first four cardiac cycles, and those observed in the abdominal aorta also disappeared. These changes were attributed to a reduction in pulmonary vascular resistance, resulting in decreased right ventricular afterload and attenuation of right-to-left shunting (27, 28). These findings suggest that oxygen supplementation may mitigate the hemodynamic consequences of both IPAVAs and right-to-left shunting through the VSD.

The diameter of the perimembranous VSD increased from 3.24 mm at the initial examination to 4.2 mm at the 5-week follow-up. Reports describing enlargement of VSDs in dogs are limited. In cattle, however, enlargement of the defect has been documented on serial examinations. Such changes have been proposed to occur in association with growth, although the underlying mechanism remains unclear. Further study is needed to clarify this phenomenon (29).

The severity of PS in dogs is commonly classified according to the PG between the RV and the pulmonary artery: mild (<50 mmHg), moderate (50–80 mmHg), and severe (>80 mmHg) (10, 24). However, Doppler-derived pulmonic velocity and PG are dependent on transvalvular flow (30, 31). At the initial examination, the left-to-right shunt through the VSD may have increased pulmonary blood flow, thereby increasing pulmonic velocity and PG (7). In contrast, the reduced TAPSE suggested impaired RV systolic function. However, interpretation of TAPSE was limited by the use of reference values established in Korean raccoon dogs with higher body weights than the Korean raccoon dog in this case (17). If RV systolic function was indeed impaired, the associated reduction in transvalvular flow may have contributed to underestimation of the Doppler-derived PG (30, 31). At the 5-week follow-up, the newly developed right-to-left shunt may have reduced pulmonary blood flow, which may have led to underestimation of the Doppler-derived pulmonic velocity and PG. Therefore, the Doppler-derived PG should be interpreted with caution when assessing the severity of PS in this Korean raccoon dog.

For dogs with severe PS, surgical or interventional treatment is generally recommended, and balloon valvuloplasty is considered the primary treatment for valvular PS (10, 32). Before intervention, coronary artery anatomy should be evaluated, as coronary anomalies may increase the risk of vascular rupture or myocardial ischemia during balloon valvuloplasty. Coronary artery anomalies can be identified by necropsy, angiocardiography, or CT angiography (9, 19). At the 5-week follow-up, the peak pulmonic velocity was 4.39 m/s, corresponding to a PG of 77.0 mmHg, consistent with moderate PS. However, considering the hemodynamic factors described above, the actual severity of PS may have been greater than indicated by Doppler measurements, and interventional treatment may still have been indicated. Accordingly, CT angiography was planned to evaluate coronary anomalies. However, the Korean raccoon dog died during anesthetic induction. Postmortem examination revealed no coronary artery abnormalities.

This case report has some limitations. First, the animal exhibited a heart rate above the reported reference range during echocardiographic examinations, likely due to excitement, which may have influenced the echocardiographic measurements (33). Sedatives and anesthetics were intentionally avoided owing to their potential effects on echocardiographic parameters (34). Second, long-term follow-up was not possible owing to the animal’s death.

To our knowledge, this case report is the first to document the coexistence of perimembranous VSD, valvular PS, and IPAVAs in a Korean raccoon dog, confirmed by echocardiography, agitated saline contrast echocardiography, and postmortem examination. The findings further suggest that progression of PS may contribute to the development of bidirectional shunting across a VSD.

## Data Availability

The raw data supporting the conclusions of this article will be made available by the authors, without undue reservation.
